# Selective impairment of affective theory of mind in restless legs syndrome: a case-control study

**DOI:** 10.1055/s-0046-1827168

**Published:** 2026-08-13

**Authors:** Aydan Topal, Suğra Şimşek, Hasan Doğan, Nazlı Durmaz Çelik

**Affiliations:** 1 Samsun University Faculty of Medicine, Department of Neurology, Samsun, Turkey.; 2 Eskişehir Osmangazi University, Faculty of Medicine, Department of Neurology, Eskisehir, Turkey.

**Keywords:** Depression, Neuropsychological Tests, Restless Legs Syndrome, Theory of Mind

## Abstract

**Background:**

Restless legs syndrome (RLS) is traditionally considered a sensorimotor disorder, but increasing evidence suggests that it may also involve brain systems related to emotional and cognitive functions. In this exploratory study, we investigated whether individuals with RLS show differences in social cognition, particularly in the ability to interpret others' emotional expressions and mental states.

**Objective:**

To investigate social cognition in individuals with RLS, particularly affective theory of mind.

**Methods:**

We compared 22 individuals with RLS and 22 healthy controls matched for age, sex, and education. Participants completed standardized tasks assessing different aspects of social cognition, including affective theory of mind, which refers to the ability to infer others' emotions.

**Results:**

We found that individuals with RLS scored lower on the task assessing affective theory of mind. No significant differences were observed in other social-cognitive measures, although the relatively modest sample size may have limited the detection of subtle differences. The group difference in affective theory of mind remained significant after accounting for depressive symptoms.

**Conclusion:**

These preliminary findings suggest that RLS may be associated with alterations in affective theory of mind independent of depressive symptoms. This pattern may reflect involvement of limbic-striatal networks. Larger studies incorporating sleep-related measures are needed to better understand social cognition in RLS.

## INTRODUCTION

Restless legs syndrome (RLS) is a chronic sensorimotor disorder characterized by unpleasant sensations accompanied by an urge to move the legs, typically worsening during rest and in the evening hours, often leading to sleep disturbance and reduced daytime functioning. 1 Although historically conceptualized within dopaminergic and sensorimotor frameworks, accumulating neurobiological evidence suggests that RLS involves multisystem dysregulation affecting broader neural networks, including dopaminergic pathways and sleep–wake regulatory circuits. 2 Clinical and neuroimaging studies have demonstrated abnormalities in thalamic, basal ganglia, and limbic-related networks in RLS, supporting the view that the disorder extends beyond peripheral sensory mechanisms. 3 4

Nonmotor features of RLS have received increasing attention due to their substantial impact on quality of life, with sleep disruption and mood symptoms being frequently reported. 5 Studies examining cognitive function in RLS have yielded heterogeneous findings: some report deficits in attention and executive control, particularly in individuals with marked sleep disturbances, whereas others observe preserved overall cognitive performance compared with matched controls. 6 These inconsistencies suggest that cognitive alterations in RLS may be subtle and domain-specific rather than global in nature.

Neurobiological models indicate that RLS involves alterations in basal ganglia–thalamocortical circuits characterized by dopaminergic, glutamatergic, and GABAergic imbalance—mechanisms with theoretical relevance for both cognitive and affective regulation. 7 8 9 10 Social cognition, encompassing processes such as theory of mind, emotion recognition, and mental-state inference, relies on distributed prefrontal, temporoparietal, and limbic networks that are sensitive to dopaminergic modulation and sleep integrity. 11 Impairments in social cognition have been documented in neurological conditions involving basal ganglia and dopaminergic dysfunction, including Parkinson's disease and Huntington's disease. 12 13 Moreover, sleep-related disorders such as obstructive sleep apnea and chronic insomnia have been associated with alterations in emotional and social-cognitive functioning. 14

Despite this convergence of neurobiological and behavioral mechanisms, social cognition has not been systematically examined in individuals with RLS, representing an important gap in understanding the broader neuropsychiatric profile of the disorder. 15 Given the interplay among dopaminergic dysregulation, sleep disturbance, and affective processing in RLS, investigating social-cognitive domains may provide insight into higher-order network involvement.

Therefore, this preliminary case–control study aimed to assess theory of mind performance and related affective measures in patients with RLS compared with matched healthy controls. We hypothesized that individuals with RLS would demonstrate altered performance on affective theory of mind measures, whereas potential differences in other social-cognitive domains might be more subtle.

## METHODS

### Participants

This preliminary case–control study was conducted at the Neurology Outpatient Clinic over a 3-month period. In total, 22 patients who met the Updated International Restless Legs Syndrome Study Group's (IRLSSG, 2014) consensus diagnostic criteria for RLS were consecutively recruited.

The control group consisted of 22 healthy volunteers, age, sex, and education-matched with the study group, recruited from hospital staff and community members. Controls were screened through clinical interviews to exclude neurological or psychiatric disorders.

Participants aged 18 years and older were eligible for inclusion. The exclusion criteria for both groups were a history of major neurological disorders (e.g., stroke, epilepsy, neurodegenerative disease), psychotic disorders, intellectual disability, substance use disorder, or any condition that could interfere with cognitive assessment.

Symptom severity was quantified using the validated Turkish version of the International RLS Rating Scale (IRLS). Dopaminergic treatment status was recorded at the time of assessment. Patients receiving treatment were using low-dose dopamine agonists; however, treatment duration and dosage were not standardized across participants.

### Measures

Social-cognitive functioning was assessed using the reading the mind in the eyes test (RMET) for affective theory of mind, the faux pas test (FPT) for cognitive theory of mind, and the facial emotion recognition test (FERT) for emotion recognition. Depressive symptoms were assessed using the Beck depression inventory (BDI).

All assessments were administered face-to-face by trained researchers in a quiet clinical setting during a single session lasting approximately 30 minutes. Standardized instructions were provided for all neurocognitive measures, and participants completed the tests individually. Due to the study design, assessors were not formally blinded to participant group.

### Ethical approval

The study protocol was approved by the institutional ethics committee (number: 2024/9/18). All procedures were conducted in accordance with the Declaration of Helsinki. Written informed consent was obtained from all participants prior to participation.

### Statistical analysis

Statistical analyses were performed using the IBM SPSS Statistics (IBM Corp.) software, version 26.0. The distribution of continuous variables was evaluated using the Shapiro–Wilk test.

Between-group comparisons for non-normally distributed continuous variables (RMET, FPT, FERT, and BDI) were conducted using the Mann–Whitney U test. Effect size for significant findings was calculated using r = Z/√N.

Associations between clinical severity (IRLS) and neurocognitive or affective measures (RMET, FPT, FERT, BDI) within the RLS group were examined using Spearman's rank correlation coefficients.

Categorical variables (age, sex, education level, and comorbidities) were compared using the Chi-squared or Fisher exact tests where appropriate.

An exploratory subgroup analysis was conducted to examine potential differences in RMET performance between patients receiving dopaminergic treatment and those not receiving treatment.

All statistical tests were two-tailed, and a *p* -value < 0.05 was considered statistically significant.

## RESULTS

A total of 44 participants were included in the study, comprising 22 patients diagnosed with RLS and 22 healthy control subjects.

The study groups' demographic and clinical characteristics are presented in Table 1 . The RLS and control groups were comparable with respect to age distribution ( *p* = 0.14), sex ( *p* = 1.00), and education level ( *p* = 0.83). No statistically significant differences were observed across these demographic variables. Within the RLS group, the mean disease severity score, as assessed by the IRLS, was 26.5 ± 4.8, indicating moderate severity.

**Table 1:** Demographic and clinical characteristics of the RLS and control groups

Variable	RLS (n = 22)	Control (n = 22)	*p* -value
Age (years)			**0.14**
< 65	16 (72.7%)	10 (45.5%)	
65–75	5 (22.7%)	8 (36.4%)	
> 75	1 (4.5%)	4 (18.2%)	
Sex			**1.00**
Female	13 (59.1%)	13 (59.1%)	
Male	9 (40.9%)	9 (40.9%)	
Schooling level			**0.83**
Primary school	4 (18.2%)	3 (13.6%)	
Secondary school	6 (27.3%)	7 (31.8%)	
High school	7 (31.8%)	8 (36.4%)	
University	5 (22.7%)	4 (18.2%)	
IRLS score (mean ± SD)	26.5 ± 4.8	–	–

**Abbreviations:** IRLS, international restless legs syndrome rating scale; RLS, restless legs syndrome; SD, standard deviation. **Notes:** Values are presented as n (%) unless otherwise indicated. No statistically significant differences were observed between groups in demographic variables.

Group comparisons of social cognition measures and depressive symptoms are presented in Table 2 . Patients with RLS demonstrated significantly lower performance on the RMET compared to healthy controls (U = 52.0, *p* < 0.001), with a large effect size (r = 0.66). No significant group differences were observed for the FERT ( *p* = 0.24) or the FPT ( *p* = 0.64), although subtle differences may not have been detectable given the modest sample size. The BDI scores also did not differ significantly between groups ( *p* = 0.78).

**Table 2:** Comparison of social cognition measures and depressive symptoms between the RLS and control groups

Variable	RLS (n = 22)	Control (n = 22)	U-value	*p* -value	Effect size (r)
RMET	18.23 ± 5.39	25.18 ± 2.58	52.0	< 0.001	**0.66**
FERT	48.43 ± 8.38	52.36 ± 3.29	182.5	0.24	0.18
FPT	67.36 ± 27.54	74.18 ± 14.94	222.0	0.64	0.07
BDI	12.73 ± 10.70	12.59 ± 9.11	229.5	0.78	0.04

**Abbreviations:** BDI, Beck Depression Inventory; FERT, Facial Emotion Recognition Test; FPT, Faux Pas Test; RMET, Reading the Mind in the Eyes Test; SD, standard deviation. **Notes:** Values are presented as mean ± SD. Group comparisons were conducted using the Mann-Whitney U test. Effect size was calculated using r = Z/√N and interpreted as small (0.1), medium (0.3), or large (0.5).

The Spearman correlation analyses were conducted to examine associations between IRLS severity and neurocognitive as well as clinical measures ( Table 3 ). The IRLS scores were not significantly correlated with RMET (ρ = -0.08, *p* = 0.71), FERT (ρ = 0.16, *p* = 0.48), or FPT scores (ρ = 0.07, *p* = 0.77). However, IRLS scores were positively correlated with BDI scores (ρ = 0.52, *p* = 0.014), indicating that greater disease severity was associated with higher levels of depressive symptoms. Importantly, depressive symptom levels did not differ between the RLS and control groups.

**Table 3:** Correlations between disease severity and neurocognitive measures in the RLS group

Variable	Spearman ρ	*p* -value
RMET	-0.08	0.71
FERT	0.16	0.48
FPT	0.07	0.77
BDI	**0.52**	**0.014**

**Abbreviations:** BDI, Beck Depression Inventory; FERT, Facial Emotion Recognition Test; FPT, Faux Pas Test; IRLS, International Restless Legs Syndrome Rating Scale; RMET, Reading the Mind in the Eyes Test. **Notes:** Spearman rank correlation coefficients are presented. Correlation analyses were conducted within the RLS group (n = 22).

An exploratory comparison revealed no significant difference in RMET performance between treated and untreated patients ( *p* = 0.53). However, this finding should be interpreted cautiously given the limited sample size and heterogeneity of dopaminergic treatment exposure within the patient group.

## DISCUSSION

The present study identified alterations in affective theory of mind in patients with RLS, as reflected by significantly lower performance on the RMET compared to healthy controls. Notably, this effect was large in magnitude, whereas no significant group differences were observed on the FERT or the FPT, although subtle differences may not have been detectable given the modest sample size. In our sample, depressive symptom levels were comparable between groups, and RMET scores were not significantly associated with disease severity or dopaminergic treatment status. These findings suggest that certain aspects of the affective theory of mind may be more susceptible to alteration in RLS.

Although RLS has traditionally been conceptualized as a sensorimotor sleep disorder, accumulating evidence supports its characterization as a network-level condition involving thalamo–striatal and limbic circuits. 1 2 3 4 5 7 These networks overlap with neural systems supporting social-cognitive processing, including medial prefrontal and amygdalo–striatal pathways implicated in rapid mental-state attribution. 8 11 In this context, the reduced RMET performance observed in our study may reflect subtle associative–limbic network involvement rather than generalized executive or perceptual impairment. At the same time, the potential contribution of sleep disturbance to affective theory of mind performance should also be considered, given the established relationship between disrupted sleep, emotional processing, and social cognition. Since sleep-related measures were not systematically assessed in the present study, the extent to which sleep disruption may have contributed to the observed RMET findings remains unclear.

The task-specific profile identified here further supports differential involvement across social-cognitive domains. While the RMET primarily assesses rapid affective mentalizing based on minimal perceptual cues, the FPT relies on structured narrative reasoning, and FERT depends on relatively robust perceptual-affective mechanisms. The preservation of FPT and FERT performance alongside reduced RMET scores suggests that rapid affective decoding may represent a particularly sensitive component within the social-cognitive architecture of RLS. Similar dissociations have been reported in disorders affecting frontostriatal circuitry, including Parkinson's and Huntington's disease, consistent with models proposing partially dissociable neural substrates for distinct social-cognitive processes. 11 12 13

Within the RLS group, disease severity was positively correlated with depressive symptom burden, indicating that greater clinical load was associated with increased affective distress. However, severity was not significantly related to RMET, FERT, or FPT performance. This pattern suggests that while mood disturbance may scale with symptom burden, the observed alteration in affective theory of mind does not appear to be a linear consequence of disease severity. Prior studies have similarly reported heterogeneous associations between RLS severity and cognitive functioning. 6 9 15

Exploratory analyses further suggested no significant association between dopaminergic treatment status and RMET performance. Although interpretation is limited by sample size and treatment heterogeneity, this observation raises the possibility that affective theory of mind alterations in RLS are not solely attributable to short-term pharmacological modulation. Longitudinal studies will be necessary to determine whether these social-cognitive features represent stable characteristics or fluctuate with disease progression and treatment dynamics.

Several limitations warrant consideration. The modest sample size limits statistical power and restricts stratified analyses. The cross-sectional design precludes causal inference, and objective sleep parameters and standardized sleep-related measures were not systematically assessed, limiting interpretation of the potential contribution of sleep disturbance to social-cognitive performance. Future investigations incorporating standardized sleep evaluation, longitudinal follow-up, and neuroimaging of associative–limbic networks will be essential to clarify the mechanisms underlying selective social-cognitive alterations in RLS.

In conclusion, the present preliminary findings suggest that patients with RLS may exhibit alterations in affective theory of mind. Although no significant differences were observed in other social-cognitive domains, subtle alterations may not have been detectable given the modest sample size. Disease severity was associated with depressive symptom burden but not with social-cognitive performance. These findings support the possibility of subtle associative–limbic involvement in RLS and highlight the need for larger studies incorporating sleep-related measures and multimodal investigation of higher-order social-cognitive processes in this population.
